# Differing effectiveness of transcranial random noise stimulation and transcranial direct current stimulation for enhancing working memory in healthy individuals: a randomized controlled trial

**DOI:** 10.1186/s12984-024-01481-z

**Published:** 2024-10-14

**Authors:** Yukina Tokikuni, Akihiro Watanabe, Hisato Nakazono, Hiroshi Miura, Ryuji Saito, Duan Miaowen, Kanako Fuyama, Keita Takahashi, Kazufumi Okada, Kazuhiro Sugawara, Harukazu Tohyama, Susumu Yoshida, Kenneth N. K. Fong, Daisuke Sawamura

**Affiliations:** 1 Graduate School of Health Sciences, Hokkaido University, Sapporo, 060–0812 Japan; 2 Department of Occupational Therapy, Faculty of Medical Science, Fukuoka International University of Health and Welfare, Fukuoka, 814-0001 Japan; 3 Data Science Center, Promotion Unit, Institute of Health Science Innovation for Medical Care, Hokkaido University Hospital, Sapporo, 060-8648 Japan; 4 Department of Physical Therapy, Sapporo Medical University, Sapporo, 060-8556 Japan; 5 Department of Rehabilitation Science, Faculty of Health Sciences, Hokkaido University, Sapporo, 060-0812 Hokkaido Japan; 6 Department of Rehabilitation Sciences, Health Sciences University of Hokkaido, Tobetsu, 061- 0293 Japan; 7 Department of Rehabilitation Sciences, The Hong Kong Polytechnic University, Hong Kong, Hong Kong

**Keywords:** Dorsolateral prefrontal cortex, Dual n-back task, Transcranial direct current stimulation, Transcranial random noise stimulation, Working memory

## Abstract

**Background:**

Transcranial direct current stimulation (tDCS) applied to the left dorsolateral prefrontal cortex (DLPFC) is a promising technique for enhancing working memory (WM) performance in healthy and psychiatric populations. However, limited information is available about the effectiveness of transcranial random noise stimulation (tRNS) applied to the left DLPFC on WM. This study investigated the effectiveness of tRNS on WM compared with that of tDCS, which has established functional evidence.

**Methods:**

This randomized, double-blind, sham-controlled trial enrolled 120 healthy right-handed adults who were randomly allocated to four stimulation groups: tRNS + direct current (DC) offset, tRNS, tDCS, or sham. Each stimulus was placed over the left DLPFC and had a current intensity of 2 mA applied for 20 min during the dual n-back task. The dual n-back task was repeated thrice: pre-stimulation, during stimulation, and post-stimulation. The d-prime scores, and response times were calculated as the main outcome measures. A linear mixed model was created to identify the main effects and interactions between the groups and times, with the group and time as fixed effects, and baseline performance and the subject as a covariate and random effect, respectively. The relationships between the benefit of each stimulus and baseline WM performance were also examined.

**Results:**

For the d-prime score during stimulation, the tRNS group significantly performed better than the sham group at online assessment (*β* = 0.310, *p* = 0.001). In the relationships between the benefit of each stimulus and baseline WM performance, the tRNS group had significantly larger negative line slopes than the sham group for the d-prime score (*β* = −0.233, *p* = 0.038).

**Conclusions:**

tRNS applied to the left DLPFC significantly improved WM performance and generated greater benefits for healthy individuals with lower WM performance. These findings highlight the potential utility of tRNS for enhancing WM performance in individuals with lower WM performance and contribute evidence for clinical application to patients with cognitive decline.

**Trial Registration:**

This study was registered in the University Hospital Medical Information Network Clinical Trial Registry in Japan (UMIN000047365) on April 1, 2022; https://center6.umin.ac.jp/cgi-open-bin/ctr/ctr_view.cgi?recptno=R000054021.

**Supplementary Information:**

The online version contains supplementary material available at 10.1186/s12984-024-01481-z.

## Background

Non-invasive transcranial electrical stimulation (tES) is a promising technique for modulating several cognitive functions in various populations, including younger and older individuals and those with diseases [1]. Among these techniques, transcranial direct current stimulation (tDCS) can modulate cortical and subcortical neuronal excitability by altering the polarity-specific membrane. Recent systematic reviews and meta-analyses have reported significant effects of tDCS on working memory (WM) performance, with an anode commonly placed over the left dorsolateral prefrontal cortex (DLPFC) in healthy and clinical populations [2–5]. The left DLPFC is a crucial brain region for WM, as commonly observed in neuroimaging studies of healthy [6, 7] and psychiatric populations [8, 9] and focal lesion studies of patients with brain injury [10–12]. Furthermore, the fronto-parietal network is a brain network in WM, involving the DLPFC and posterior parietal cortex [13]. The roles of these local and intracerebral networks and their relationships vary depending on the task content and difficulty of WM [14–16].

Combined or multimodal treatments of cognitive training and tDCS have shown promise and are receiving increasing attention as cognitive intervention strategies for enhancing WM in individuals with cognitive decline. However, the observed effect sizes are typically small and heterogeneous [2–5]. Moreover, null findings for the effects of tDCS on WM have also been reported [17, 18], indicating that the evidence is not conclusive.

Transcranial random noise stimulation (tRNS) is an emerging non-invasive neuromodulation technique characterized by alternating currents delivered at random intensities. Its effectiveness was first reported in 2008 [19] and has since received increasing scientific interest. tRNS has some benefits over other tES methods, including reduced discomfort, more successful blindness, and larger neuromodulating effects on neurophysiological and behavioral outcomes [20–22].

The underlying neurophysiological mechanism of neuromodulation with tRNS remains unclear, although stochastic resonance has been proposed as the representative underlying mechanism of tRNS in several studies [23, 24]. Stochastic resonance is a phenomenon in which an optimal level of noise enhances weak signal detection [1]. At the neuronal level, stochastic resonance can occur when excitatory and inhibitory postsynaptic potentials are combined with exogenous polarizing mechanisms [25]. tRNS can also be applied with a direct current (DC) offset, which produces a unidirectional current flow analogous to tDCS (polarization of the membrane potential) by combining the characteristics of tDCS and tRNS (potentially introducing noise into the neural system) [26]. The tRNS frequency band ranges from 0.1 to 640 Hz and can be classified as low frequency (0.1–100 Hz) and high frequency (101–640 Hz), as previously determined by applying a 640 Hz frequency [19], which is the high end of physiologically measured human brain oscillations [27]. The greater neuromodulation of high-frequency tRNS has shown neurophysiological effects in different brain regions (primary [19, 28], prefrontal [22, 29, 30], auditory [31], parietal [32], and visual [33] cortexes) and motor [19, 28], sensory, perception [23, 24, 34], and cognitive processes (attention [30, 35], WM [20], learning [36, 37], arithmetic ability [29], and face memory performance [38]).

However, the efficacy of this technique remains unclear. Some studies have investigated the effects of tRNS paired with WM tasks and reported varied effects [22, 39, 40]. Furthermore, to the best of our knowledge, only two studies have directly compared the effects of tRNS and tDCS on WM performance [22, 39]. Mulquiney et al. [39] investigated the effects of 10-min tRNS applied to the left DLPFC on the n-back task (1-back and 2-back tasks) in three groups: tRNS, tDCS, and sham stimulation; the results showed that tDCS alone significantly improved performance speed. Murphy et al. [22] delivered 1 mA tRNS with a 1 mA DC offset over the left DLPFC for 20 min during the Paced Auditory Serial Addition Task (PASAT); the results showed a more pronounced and consistent improvement in performance of the Sternberg WM task than in the tDCS and sham tasks. Thus, these two studies did not reach a unanimous conclusion, although differences in tRNS parameters may have contributed to their inconsistency. Mulquiney et al. [39] applied tRNS without a DC offset, whereas Murphy et al. [22] delivered a tRNS with DC offset. These findings suggest that future studies should examine the effects of different types of tRNS stimuli.

This prospective study aimed to investigate the effectiveness of tRNS with and without a DC offset and tDCS to the DLPFC on WM performance. We hypothesized that tRNS would have a more beneficial effect on WM performance than tDCS or sham treatment. Considering the clinical application for cognitive decline, psychiatric disorders, and other brain disorders, we selected the dual n-back task (DBT) as a WM task because there is some evidence of its beneficial effects in reducing aberrant brain activity and improving cognitive function in these populations [41–44]. This study can contribute to cognitive rehabilitation for enhancing WM performance in individuals with cognitive decline and more effective combined and multimodal treatments using tES in clinical populations with brain injury and neurodegenerative and psychiatric disorders.

## Methods

### Trial design and participants

This was a double-blind, randomized, sham-controlled trial, stratified by sex. A total of 120 right-handed healthy adults participated during the 9-month study period (July 2022 to March 2023). Handedness was evaluated using the Edinburgh Handedness Inventory (EHI) [45]. The sample size required was estimated using G power 3.1 [46] based on two studies that investigated the effects of tRNS on WM [20, 37]. We adopted partial *η*^*2*^ (*η*^*2*^_*p*_) based on a mixed-design analysis of variance (ANOVA) model for the given effect sizes because linear mixed models (LMMs) do not provide these respective values. The sample size estimate for time × group interaction was based on a significance level (*α*) of 0.05, statistical power of 0.95, and given effect size (*f)* of 0.176 calculated by the effect size of time × group interaction *η*^*2*^_*p*_ of 0.03 [39], resulting in a suggested *n* of 100. To ensure a conservative estimation, 20% was added to account for possible dropouts or outliers, with the sample size (*n*) set at 120.

**Fig. 1:**
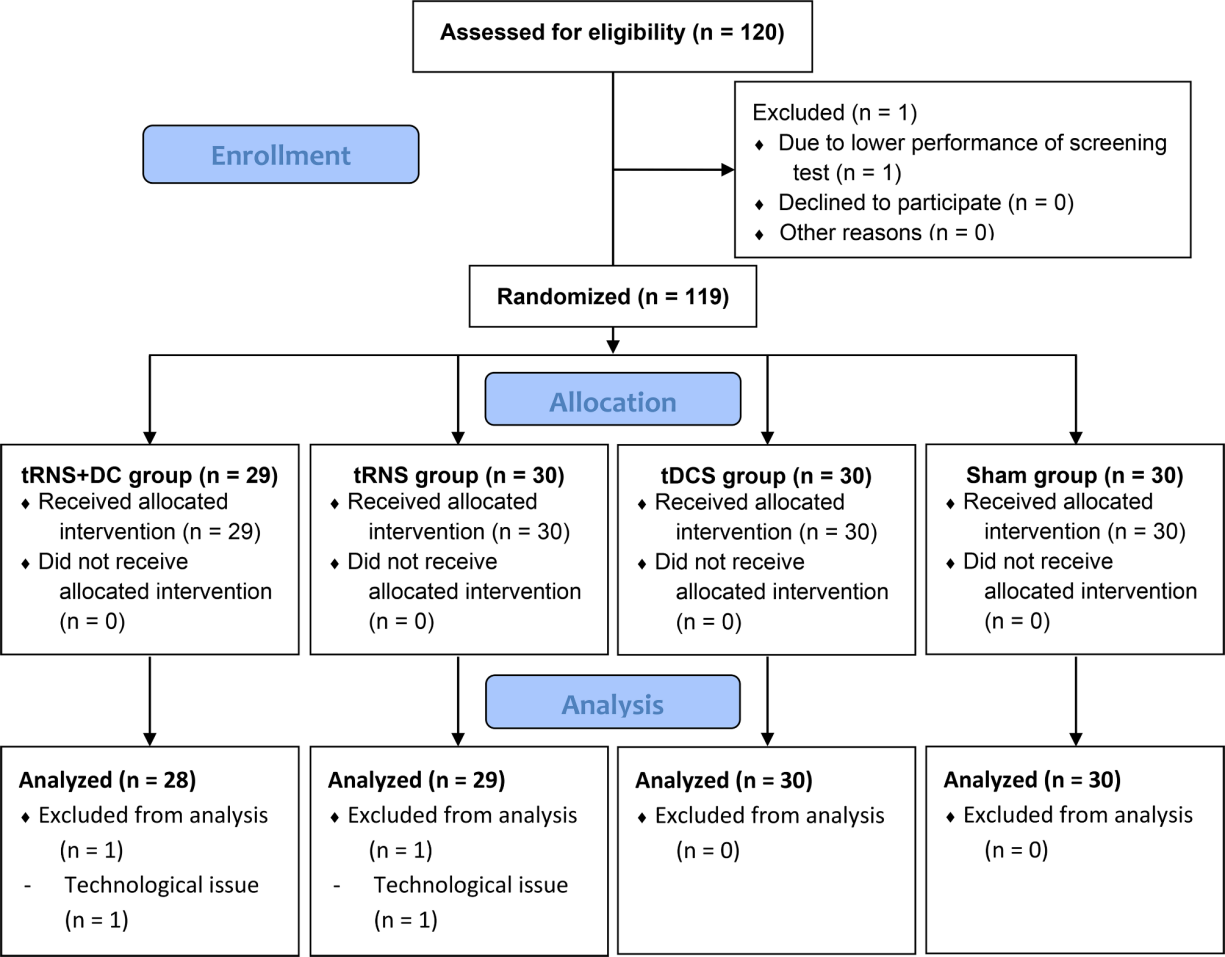
CONSORT flow diagram. Flow diagram with the number of participants and demonstrating the procedural steps to the final data analyses. DC: direct current, tDCS: transcranial direct current stimulation, tRNS: transcranial random noise stimulation

The inclusion criteria were participants aged 18–30 years with no experience performing DBT. The exclusion criteria were a history of neurological or psychiatric disorders, low performance on the screening tests (defined as scores lower than mean − 2 SD, as demonstrated by a previous report [47], observed in any of the following screening tests: digit span test forward < 6 and backward < 5, tapping span test forward < 5 and backward < 4), insufficient safety assessed using a safety questionnaire for TES (history of epilepsy, intracranial ferromagnetic metal implants, and pregnancy) [48], and an EHI score of < 70 points. One participant was excluded because of poor performance in the screening test (Fig. 1).

Independent researchers randomly allocated all participants to the tRNS + DC offset (*n =* 29; 14 women), tRNS (*n =* 30; 15 women), tDCS *(n =* 30; 15 women), or sham (*n =* 30; 15 women) groups, according to a computer-generated randomization list. All participants and experimenters not involved in randomization were blinded to the brain stimulation assignments.

All experiments were conducted in accordance with the Declaration of Helsinki. Written informed consent was obtained from all participants. This study was approved by the Ethics Committee of the Faculty of Health Sciences at Hokkaido University (approval number: 22 − 6) and registered in the University Hospital Medical Information Network Clinical Trial Registry in Japan (UMIN000047365). This study was conducted in accordance with the Consolidated Standards of Reporting Trials guidelines.

### Experimental procedure

**Fig. 2:**
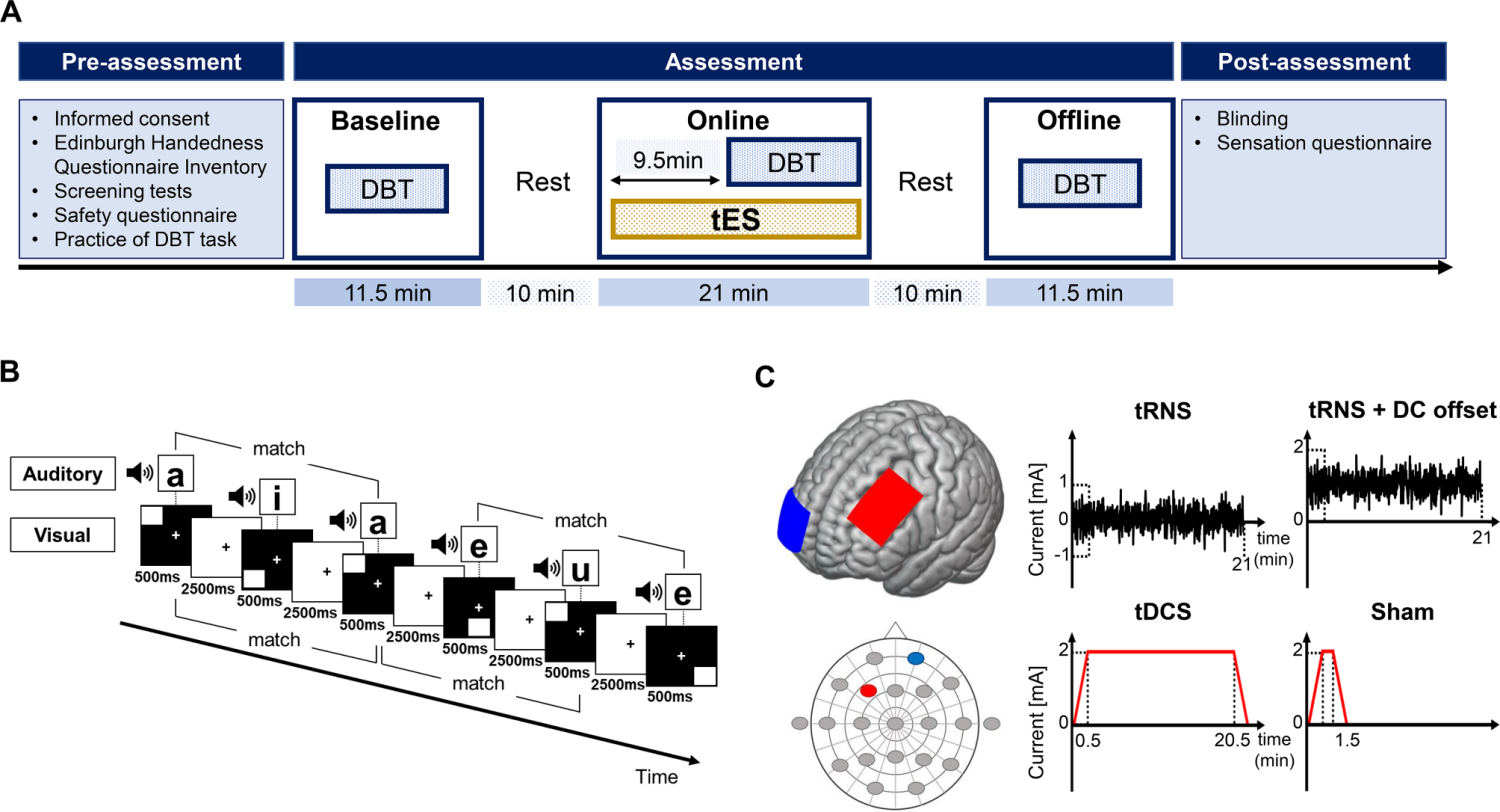
Schematic of the experimental procedure, task, and transcranial electrical stimulation. **(A)** Overview of experimental procedure. The experimental procedure comprised three phases: pre-assessment, assessment, and post-assessment. In the assessment phase, the dual n-back task was performed for 11.5 min in each session, and transcranial electrical stimulation was delivered for 21 min in the online session. **(B)** Dual n-back task. The series of auditory and visuospatial stimuli are presented simultaneously. Participants were required to quickly answer whether the current stimulus was the same as the stimulus presented two times before. **(C)** Electrode configurations and four types of electrical current waveforms. Anode and cathode are respectively placed over the left DLPFC (F3) and the right orbitofrontal cortex (Fp2) according to the international 10/20 system. While tDCS delivers a direct electrical current with a constant intensity, tRNS delivers an alternating current with a random frequency and intensity. The vertical axis illustrates the current intensity, and the horizontal axis illustrates the time-course. DBT: dual n-back task, DC: direct current, tDCS: transcranial direct current stimulation, tES: transcranial electrical stimulation, tRNS: transcranial random noise stimulation

The experimental procedure included three distinct phases: pre-assessment, assessment, and post-assessment (Fig. 2A). All procedures were performed on a single day. In the pre-assessment phase, participants completed the EHI and safety questionnaires. Subsequently, the participants underwent screening tests, and the experimental procedure was explained. Finally, the participants were asked to practice DBT for 2.5 min, comprising 50 trials, to help them understand how to perform it and minimize the learning effect.

During the assessment phase, all participants completed DBT at three assessment periods: baseline, during tES (online), and post-tES (offline) (Fig. 2A). The four types of stimulations comprised tRNS + DC offset, tRNS, anodal tDCS, and sham. All types of left DLPFC stimulation were administered. Participants were informed that they would receive “forehead stimulation of two different intensities” and were blinded to the stimulation mode; they performed four blocks of the DBT in each assessment period, with 150 s task and 30 s inter-task intervals, for a total duration of 11.5 min (Fig. 2A). Each assessment phase was separated by a 10 min rest period. For the initial online 9.5 min, the participants sat on a chair and received stimulation during rest. To ensure thorough experimental blinding, each independent investigator played a separate role in assessing DBT performance or delivering the stimuli. Other experimenters were responsible for the pre- and post-assessment procedures.

In the post-assessment phase, to determine the effectiveness of stimulation in improving DBT task performance, stimulation blindness was assessed by asking participants to guess whether they received active or sham stimulation during the post-stimulation phase. Subjective discomfort was determined using a sensation questionnaire to assess participant safety and tolerability [49].

### DBT

DBT was created using E-prime ver. 3.0 (Psychology Software Tools, Pittsburgh, PA, USA) (Fig. 2B). Visuospatial and auditory WM tasks employed by Jaeggi et al. [50] were used. The visuospatial task was randomly presented a 3 × 3 square at eight different locations on the computer screen. The auditory task randomly presented one of the Japanese kana phonetic characters (“a,” “i,” “u,” “e,” or “o”). A series of visuospatial and auditory stimuli were synchronously and sequentially presented simultaneously for 3,000 ms per stimulus, which comprised a 500-ms stimulus duration and 2,500-ms interstimulus interval. The audio volume was set to 70 dB for participants to clearly hear the auditory stimuli. In this task, the participants were required to quickly answer by pressing a key (auditory “A” and visual “L”) on the keyboard when the current stimulus was the same as the stimulus presented two times before. Each DBT block comprised 50 stimuli (auditory target: 10 stimuli [20%], visual target: 10 stimuli [20%], audiovisual target: 5 stimuli [10%], no target: 25 stimuli [50%]) for 2.5 min, and four blocks were performed. These proportions of stimulus targets were determined with reference to previous studies [47]. Data from the first DBT were removed from the analysis because of the greater learning effect from the first to the second DBT. The order of stimulus presentation was randomized for each DBT. The d-prime scores (d‘s), and response times were calculated as the main performance indicators. d‘ is a measurement from signal detection theory that measures the distance between signal and noise in standard deviation units and is calculated as follows: d’ = z (hit rate) − z (false alarm rate). The participants did not receive any guidance or feedback throughout the experiment to ensure the net tDCS impact.

### Transcranial electrical stimulation

The four types of stimulation, tRNS + DC offset, tRNS, tDCS, and sham stimulation, were applied using the DC-STIMULATOR PLUS (NeuroConn GmbH, Ilmenau, Germany) with a pair of 0.9% saline-soaked 5 × 7 cm rubber electrodes, resulting in a current density of 0.057 mA/cm^2^, which is well within the current safety criteria [51]. For all stimulation conditions, a pair of electrodes was placed on the left DLPFC (F3 in the international 10–20 electroencephalography [EEG] system) and the right supraorbital cortex (Fp2) using two circumferential straps. A schematic of the electrode configuration and all stimulations is presented in Fig. 2C.

The electrode configuration was determined based on prior neuroimaging studies that demonstrated increased activation during WM [52–54] and on prior neuromodulation studies that demonstrated improved WM performance [55]. In addition, a previous meta-analysis reported that higher-intensity and longer-duration tDCS over the left DLPFC is more effective at enhancing cognitive function in healthy and clinical populations [5]. Moreover, studies have reported that 2 mA anodal tDCS for 20 min is effective in increasing cortical excitability and generating long-lasting effects [56, 57]. As the effect of tDCS was used for comparison with evidence for tRNS + 1 mA DC offset and tRNS, the same current intensity (2 mA for a duration of 20 min) was commonly employed for all stimulus types to match the established evidence for tDCS on WM performance. High-frequency tRNS (100–640 Hz) was delivered at 2 mA (current intensity range − 1.0 to 1.0 mA), with and without a DC offset of 1 mA. A high-frequency range within the upper end of physiologically measured human brain oscillations (100–640 Hz) [27] was selected based on the results of previous studies that demonstrated greater neuromodulatory effects relative to a low-frequency tRNS (0–100 Hz) [19, 36]. The tRNS + DC offset with these parameters produces an alternative current flow analogous to tDCS and ensures that each electrode consistently maintains polarity [26]. The tRNS + DC offset current in this study flowed from the positively charged anode (current intensity range 0–2.0 mA) to the negatively charged cathode (current intensity range 0 to − 2.0 mA).

In the three stimulation types, except sham stimulation, a 30 s gradual ramping up to 2 mA and 30 s gradual ramping down were adopted before and after the 20 min online session. In the sham group, 30 s of gradual ramping up to 2 mA, 15 s of 2 mA stimulation, and 30 s of gradual ramping down were employed during the first 75 s of the 20 min session, with 0 mA used for the remainder of the period. Impedances were maintained below 5.0 kΩ under all stimulation conditions.

### Statistical analyses

Baseline characteristics, sensation questionnaires, and blinding assessment were analyzed using one-way ANOVA, Kruskal–Wallis test, or Chi-square test.

Two outcome measures (d’ and response time) were introduced into an LMM after confirming normality using the Shapiro–Wilk test. Although the Shapiro–Wilk test for normality showed a significance in only one of 24 distributions comprising four stimulation types, two measurements (d’, and response time) and three assessment sessions (baseline, online, and offline), all of the skewness statistics lay between − 1.96 and 1.96, which does not indicate a substantial deviation from the normality [58, 59]. Accordingly, LMM was performed in all of these distributions. In the LMM, stimulation type (tRNS + DC offset, tRNS, tDCS, and sham), time period (online and offline), and their interactions were included as fixed effects. Baseline performance and participant identification were included as a covariate and random effect, respectively. The Akaike information criterion was used to assess the goodness of fit of the candidate models. Participant identification, a random effect, was included in the model if it did not increase the Akaike information criterion by > 5 points [60]. In addition, to characterize the beneficial effect of each stimulus that was dependent on baseline WM performance, a general linear model was used for each outcome measure to detect the differences in the regression line slope of the three stimulation groups compared with the sham group.

All statistical analyses were performed using SPSS version 26.0 for Windows (IBM Corp., Armonk, NY, USA). Statistical significance was set at 0.05.

## Results

### Demographics, sensation questionnaire, and blinding after stimulus application

Figure 1 presents the analysis flow diagram. The final sample comprised 117 right-handed healthy young adults (aged 18–28 years; mean age 22.56 ± 2.13 years; mean EHI score 96.65 ± 7.12). Table 1 presents information on demographics, sensation questionnaires, and blinding.

**Table 1:** Demographics and assessments of screening, blinding, and sensation

	tRNS + DC *(n =* 28*)*	tRNS *(n =* 29*)*	tDCS *(n =* 30*)*	Sham *(n =* 30*)*	*p*-value	Statistics
Participant demographics						
Age years; mean (SD)	22.3 (2.42)	23.1 (2.05)	22.3 (2.12)	22.5 (1.96)	0.406	*F* = 0.977
Sex						
Males (*n* %)	15 (53.6)	15 (51.7)	15 (50.0)	15 (50.0)	0.992	*χ²* = 0.100
Females (*n* %)	13 (46.4)	14 (48.3)	15 (50.0)	15 (50.0)		
Edinburgh Handedness Inventory Mean (SD)	92.5(9.57)	98.7 (3.87)	97.2 (6.46)	97.9 (6.34)	0.004**	*F* = 4.774
Education Mean (SD)	15.5 (1.72)	16.6 (1.63)	15.6 (1.55)	15.8 (1.49)	0.061	*F* = 2.529
Screening test						
Digit span Forward mean (SD)	7.25 (0.97)	7.34 (1.04)	7.37 (0.81)	7.17 (1.09)	0.696	*H* = 1.441
Digit span Backward mean (SD)	5.79 (1.03)	5.97 (1.09)	5.80 (1.16)	6.13 (1.31)	0.731	*H* = 1.291
Tapping span Forward mean (SD)	7.11 (1.17)	7.24 (1.27)	6.90 (0.92)	6.97 (1.07)	0.552	*H* = 2.098
Tapping span Backward mean (SD)	6.54 (1.35)	7.38 (1.12)	6.57 (1.14)	6.83 (1.23)	0.059	*H* = 7.448
Blinding						
Felt stimulated *n* (%)	17 (60.7)	11 (37.9)	15 (50.0)	19 (63.3)	0.195	*χ²* = 4.707
Felt non-stimulated *n* (%)	11 (39.3)	18 (62.1)	15 (50.0)	11 (36.7)		
Sensation questionnaire Felt stimulated (%)						
Tingling pain	0.0 (0.0–1.0)	0.0 (0.0–0.0)	0.0 (0.0–1.0)	0.0 (0.0–1.0)	0.003**	*H* = 13.930
Pain	0.0 (0.0–0.0)	0.0 (0.0–0.0)	0.0 (0.0–0.0)	0.0 (0.0–0.0)	0.244	*H* = 4.171
Burning	0.0 (0.0–0.0)	0.0 (0.0–0.0)	0.0 (0.0–0.0)	0.0 (0.0–0.0)	0.288	*H* = 3.766
Warmth/Heat	0.0 (0.0–0.0)	0.0 (0.0–0.0)	0.0 (0.0–0.0)	0.0 (0.0–0.0)	0.365	*H* = 3.175
Metallic	0.0 (0.0–0.0)	0.0 (0.0–0.0)	0.0 (0.0–0.0)	0.0 (0.0–0.0)	0.561	*H* = 2.057
Fatigue	0.0 (0.0–0.0)	0.0 (0.0–0.0)	0.0 (0.0–0.0)	0.0 (0.0–1.0)	0.064	*H* = 7.248
Others	0.0 (0.0–0.3)	0.0 (0.0–0.0)	0.0 (0.0–1.0)	0.0 (0.0–0.0)	0.014*	*H* = 10.606

DC: direct current, SD: standard deviation, tDCS: transcranial direct current stimulation, tRNS: transcranial random noise stimulation. *: *p* < 0.05, **: *p* < 0.01

There were significant differences in EHI scores, indicating a lower EHI score in the tRNS + DC group than in the tRNS and sham groups. In addition, significant differences among the four groups were demonstrated in the sensational questionnaire, tingling pain, and others, indicating a higher tingling pain score in the tDCS group than in the tRNS group. However, we did not consider including these variables as covariates in the LMM because despite differences in scores, successful blinding and right-handedness were ensured, and their impact on outcome measures was considered negligible. No significant differences among the groups were observed for the other variables (Table 1).

### DBT performance

**Fig. 3:**
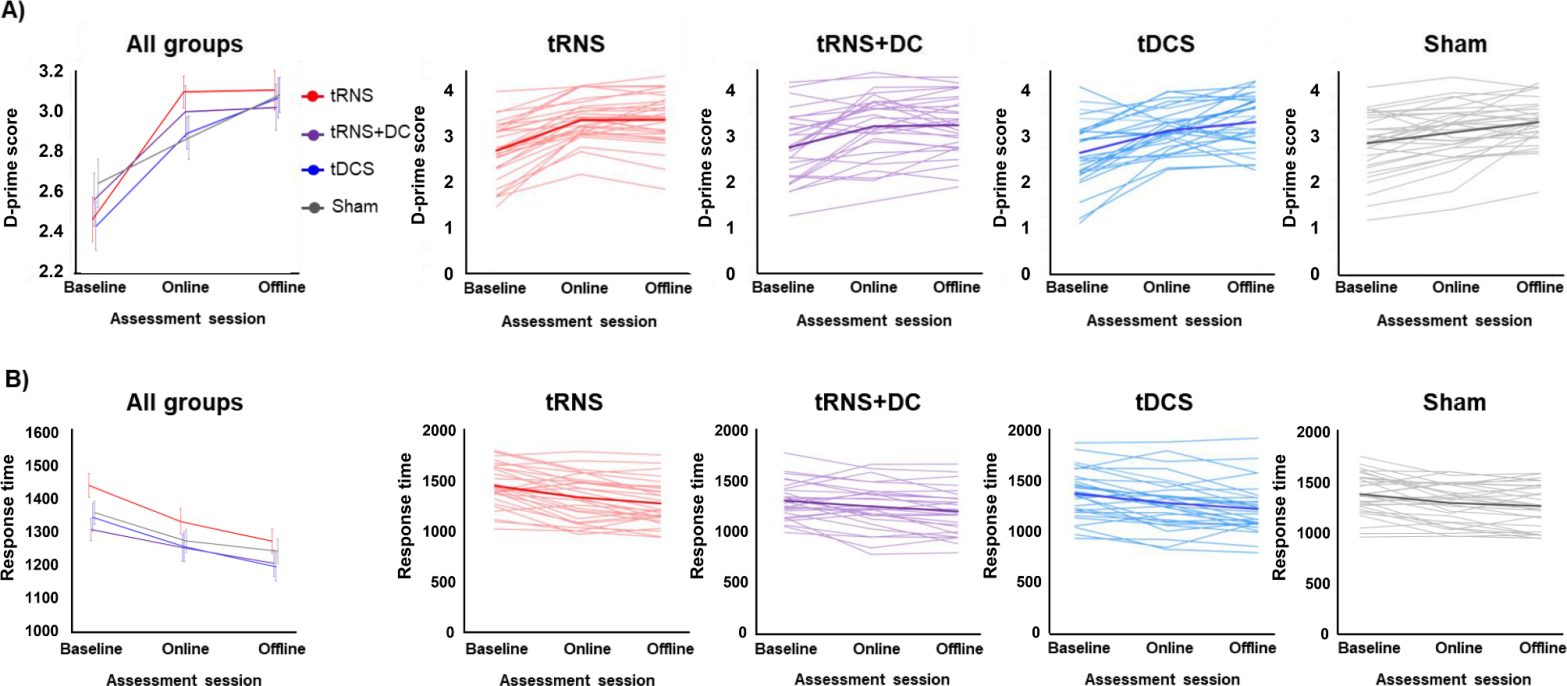
Group performance in dual n-back task across two assessment sessions (baseline, online, offline sessions). **(A)** D-prime score, **(B)** Response time. All left figures show mean Z-scores and standard errors in all groups, others show the individual performance of the dual n-back task in each of the four stimulation groups. The bold line shows the mean z-score of each group performance. DC: direct current, tDCS: transcranial direct current stimulation, tRNS: transcranial random noise stimulation

Figure 3 presents the two outcome measures, d’ and response time, at two assessment sessions in all groups.

Regarding d’, there were significant effects of stimulation type in the tRNS group compared with that in the sham (*β* = 0.310, 95% confidence interval [CI] = [0.128, 0.492], t[166.8] = 3.289, *p* = 0.001) and time in the offline assessment compared with that for online (*β* = 0.202, 95% CI = [0.08, 0.319], t[113] = 3.346, *p* = 0.001) (Table 2). In addition, significant stimulation type × time interactions were observed in the tRNS + DC × offline and tRNS × offline (tRNS + DC× offline: *β* = 0.202, 95% CI = [0.082, 0.321], t[113] = 3.346, *p* = 0.001, tRNS × offline: *β* = 0.173, 95% CI = [0.054, 0.292], t[113] = 2.870, *p* = 0.005). Pairwise contrasts of stimulation only showed a significant difference between tRNS and sham in the online session (*β* = 0.310, 95% CI = [0.123, 0.496], t[167] = 3.279, *p* = 0.001), indicating significantly better performance in the tRNS group (Table 3). In addition, significant differences between online and offline sessions were observed in the sham and tDCS groups (Sham: *β* = 0.202, 95% CI = [0.082, 0.321], t[113] = 3.346, *p* = 0.001, tDCS: *β* = 0.173, 95% CI = [0.054, 0.292], t[113] = 2.870, *p* = 0.005).

**Table 2:** Estimated regression coefficients of the linear mixed model for d-prime score

	Estimate	SE	df	95% CI		t	*p*
				Lower	Upper		
Intercept	1.281	0.140	122.8	1.011	1.552	9.147	< 0.001***
Baseline of d-prime score (per 1)	0.610	0.046	112.0	0.520	0.699	13.131	< 0.001***
Stimulation (ref. = sham)							
tRNS + DC	0.155	0.095	167.1	-0.029	0.338	1.627	0.105
tRNS	0.310	0.094	166.8	0.128	0.492	3.279	0.001**
tDCS	0.127	0.094	166.6	-0.054	0.308	1.357	0.177
Time (ref. = online)	0.202	0.060	113	0.084	0.319	3.346	0.001**
Interaction							
tRNS + DC*Offline	0.182	0.087	113	-0.350	-0.013	–2.094	0.038*
tRNS*Offline	0.193	0.086	113	-0.360	-0.026	-2.245	0.027*
tDCS*Offline	0.029	0.085	113	-0.194	0.136	-0.336	0.737

CI: confidence interval, DC: direct current, *df*: degrees of freedom, SE: standard error, tDCS: transcranial direct current stimulation, tRNS: transcranial random noise stimulation. *: *p* < 0.05, **: *p* < 0.01, ***: *p* < 0.001

**Table 3:** Contrasts of the linear mixed model for d-prime score

	Estimate	SE	df	95% CI		t	*p*
				Lower	Upper		
Stimulation (Online)							
tRNS + DC – sham	0.155	0.095	167	–0.033	0.342	1.627	0.106
tRNS – sham	0.310	0.094	167	0.1233	0.496	3.279	0.001**
tDCS – sham	0.127	0.094	167	-0.058	0.313	1.357	0.177
tRNS + DC – tDCS	0.027	0.095	167	-0.160	0.215	0.287	0.774
tRNS – tDCS	0.183	0.094	167	-0.003	0.369	1.934	0.060
tRNS + DC – tRNS	-0.155	0.096	167	-0.344	0.034	-1.619	0.107
Stimulation (Offline)							
tRNS + DC – sham	-0.027	0.095	167	-0.215	0.161	-0.284	0.777
tRNS – sham	0.116	0.095	167	-0.069	0.303	1.237	0.218
tDCS – sham	0.099	0.094	167	-0.087	0.284	1.051	0.295
tRNS + DC – tDCS	-0.126	0.095	167	-0.141	0.235	1.320	0.189
tRNS – tDCS	0.018	0.094	167	-0.168	0.204	0.193	0.847
tRNS + DC – tRNS	-0.144	0.096	167	-0.333	0.046	-1.500	0.136
Time (Offline – Online)							
sham	0.202	0.060	113	0.321	0.082	3.346	0.001**
tRNS + DC	0.020	0.062	113	-0.104	0.144	0.321	0.749
tRNS	0.009	0.061	113	–0.444	0.443	0.141	0.888
tDCS	0.173	0.060	113	0.054	0.292	-2.870	0.005**

CI: confidence interval, DC: direct current, *df*: degrees of freedom, SE: standard error, tDCS: transcranial direct current stimulation, tRNS: transcranial random noise stimulation. *: *p* < 0.05, **: *p* < 0.01

For response time, no significant main effects of stimulation type and time, and interactions were observed (Table 4). In the pairwise contrasts, significant differences were only observed in the time (offline – online) in all groups, excluding the sham group (Table 5).

**Table 4:** Estimated regression coefficients of the linear mixed model for response time

	Estimate	SE	df	95% CI		t	*p*
				Lower	Upper		
Intercept	133.045	115.498	1.603	-27.388	293.477	0.112	0.342
Baseline of d-prime score (per 1)	0.833	0.059	112.0	0.719	0.946	14.188	< 0.001***
Stimulation (ref. = sham)							
tRNS + DC	35.846	37.574	151.9	-36.697	108.388	0.954	0.342
tRNS	4.457	37.407	151.5	-67.763	76.677	0.119	0.905
tDCS	-0.833	36.792	152.2	-71.866	70.201	-0.023	0.981
Time (ref. = online)	-21.104	20.582	113	-61.076	18.868	-1.025	0.307
Interaction							
tRNS + DC*Offline	-32.794	29.622	113	-90.323	24.735	-1.107	0.271
tRNS*Offline	-39.149	29.357	113	-96.163	17.864	-1.334	0.185
tDCS*Offline	-35.735	29.107	113	-92.264	20.794	-1.228	0.222

CI: confidence interval, DC: direct current, *df*: degrees of freedom, SE: standard error, tDCS: transcranial direct current stimulation, tRNS: transcranial random noise stimulation. ***:* p* < 0.001

**Table 5:** Contrasts of the linear mixed model for response time

	Estimate	SE	df	95% CI		t	*p*
				Lower	Upper		
Stimulation (Online)							
tRNS + DC – sham	35.846	37.6	152	–38.4	110.1	0.954	0.342
tRNS – sham	4.457	37.4	152	-69.4	78.4	0.119	0.905
tDCS – sham	-0.833	36.8	152	-73.5	71.9	-0.023	0.982
tRNS + DC – tDCS	36.679	37.5	152	-37.4	110.8	0.978	0.330
tRNS – tDCS	5.289	37.5	151	-68.8	79.4	0.141	0.888
tRNS + DC – tRNS	31.389	38.6	150	–44.9	107.6	0.813	0.417
Stimulation (Offline)							
tRNS + DC – sham	3.052	37.6	152	–71.2	77.3	0.081	0.935
tRNS – sham	-34.693	37.4	152	-108.6	39.2	-0.927	0.355
tDCS – sham	-36.568	36.8	152	-109.3	36.1	-0.994	0.322
tRNS + DC – tDCS	39.620	37.5	152	-34.5	113.7	1.056	0.293
tRNS – tDCS	1.875	37.5	151	-72.3	76.0	0.050	0.960
tRNS + DC – tRNS	37.744	38.6	150	–38.5	114.0	0.978	0.330
Time (Offline – Online)							
sham	-21.104	20.6	113	–61.9	19.7	-1.025	0.307
tRNS + DC	-53.899	21.3	113	-96.1	-11.7	-2.530	0.013*
tRNS	-60.254	20.9	113	–101.7	-18.8	-2.878	0.005**
tDCS	-56.840	20.6	113	–97.6	-16.1	-2.762	0.007**

CI: confidence interval, DC: direct current, *df*: degrees of freedom, SE: standard error, tDCS: transcranial direct current stimulation, tRNS: transcranial random noise stimulation. *: *p* < 0.05, **: *p* < 0.01

The statistical observation of the results separately for males and females was shown in supplementary material 1. There were no significant differences in d’ and response time between them in any stimulation types and assessment sessions.

### Relationships between baseline performance and changes in performance for each assessment period

General linear models were used for each outcome measure to detect the differences in the regression line slope of the three stimulation types compared with the sham, which aids in elucidating whether each tES had a characteristic effect that was dependent on the WM performance and providing valuable information for future clinical application.

**Fig. 4:**
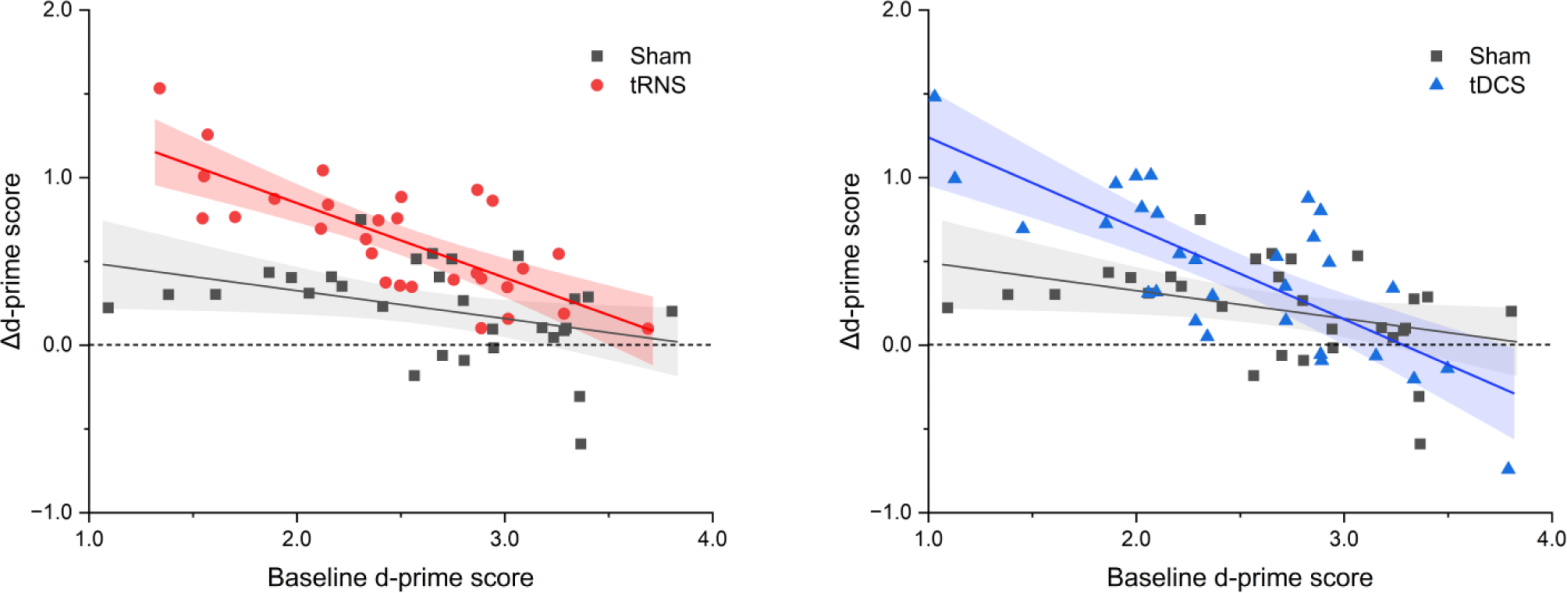
Different line slopes in linear regressions with interactions. **(A)** tRNS vs. sham for online changes in the d-prime score of DBT. **(B)** tDCS vs. sham for online changes in the d-prime score of DBT. The straight and curved lines show the mean and 95% confidence interval, respectively. DBT: dual n-back task, tDCS: transcranial direct current stimulation, tRNS: transcranial random noise stimulation

For d’, there was a significant stimulation type (tRNS vs. sham and tDCS vs. sham) × baseline performance interaction for online changes, indicating a significantly steeper negative slope in tRNS and tDCS groups than in the sham group (tRNS: *β* = −0.233, 95% CI = [− 0.452, − 0.129], t [55] = − 2.122, *p* = 0.038, tDCS: *β* = −0.358, 95% CI = [− 0.590, − 0.127], t [56] = − 3.099, *p* = 0.003) (Fig. 4). No other significant interactions for online and offline changes in pairs of stimulus types was observed for any of the two outcome measures (all *p* > 0.05).

## Discussion

This prospective, hypothesis-driven, double-blind, sham-controlled, randomized controlled study fulfilled the criteria suggested in a recent consensus and critical position article on tDCS [61] because transparency, reproducibility, and standardization were considered. To the best of our knowledge, this is the first study to evaluate the effects of three types of tES applied to the left DLPFC for enhancing WM during and after stimulation. The results indicated the success of the blinded questionnaire evaluation despite differences in subjective discomfort (“tingling pain” and “others” had higher scores in tDCS than in other stimulations). This reinforces the justification of our findings.

We found that tRNS targeting the left DLPFC during stimulation only improved WM performance. Specifically, tRNS to the left DLPFC significantly improved the d-prime score compared with sham during stimulation. Although there were no significant changes in response time, it is still worth noting that there was no trade-off between tRNS effects on response time and d-prime score. These results are compatible with those of reports that showed less discomfort and larger neuromodulating effects on neurophysiological and behavioral outcomes with tRNS than with tDCS [20, 21, 62]. However, no significant differences were observed between the active stimulation types. The only higher d-prime score was observed in tRNS compared to that in tDCS, a traditional representative technique, tended to be significant. While tDCS has beneficial effects on WM performance, the high variability in its effects across studies and even at an individual level has been reported in several meta-analyses [5, 17, 63]. Consistent with these findings, a high variability in the effect of tDCS was observed in this study. While the other groups showed 0–10% of cases of lower performance at the online and offline sessions compared to that at baseline, the DCS group showed that 20% and 16.7% of participants had lower performance at online and offline sessions compared to that at baseline, respectively. Moreover, a few neuroimaging studies on the neuromodulating effects of resting-state connectivity using tDCS applied to the left DLPFC reported inconsistent results in the frontoparietal network involved in WM [64, 65]. Taken together, these findings suggest that the null findings of tDCS are largely consistent with those in previous studies [17, 18], suggesting that it might not be sufficient to generate meaningful or consistent performance gains in WM in healthy adults.

A recent review reported the effectiveness of tRNS for perception and cognitive function, although it only reported insufficient evidence on WM [25]. Our results differ from previous findings that suggested no effect of high-frequency tRNS without DC offset over the left DLPFC on WM performance. Several methodological factors may have contributed to these conflicting findings. First, a weaker intensity (1.0 mA) was commonly used in previous studies [39, 40]. A recent neurophysiological study reported a gradual increase in motor-evoked potential for higher tRNS intensities during stimulation among different intensities (0.5, 1.0, 1.5, and 2.0 mA), indicating that 2 mA stimulation is most effective for boosting cortical excitability [66]. Although the targeted brain region is different, it is possible that similar higher cortical excitability was induced in 2 mA tRNS to the DLPFC and resulted in improved WM performance.

Second, the selected WM tasks differed. One study selected the Sternberg WM task, and another adopted multiple WM tests with a focus on span tasks.

The Sternberg WM task and multiple WM tests strongly reflect aspects of WM capacity and are different from DBT, which includes aspects of executive control of WM, such as divided attention, a higher load of information processing and memory updating, simultaneous task performance, updating necessary information, and quickly eliminating unnecessary information. Moreover, DBT requires a higher cognitive demand than these tasks. Previous neuromodulation studies targeting the left DLPFC reported that its effectiveness is robust in high cognitive demands for a target task [67, 68]. These findings may strengthen the validity of our results.

Only one study [22] has reported the successful effects of tRNS on WM and its underlying neurophysiological evidence in healthy participants. This study adopted the Sternberg WM task and delivered 1 mA tRNS with a 1 mA DC offset over the left DLPFC (cathode over the contralateral supraorbital) during PASAT, which engages the frontoparietal network involved in WM processing. We evaluated the effects of tRNS and tDCS on WM function and oscillatory power, as assessed using EEG. Murphy et al. [22] found that tRNS with a 1 mA DC offset had more beneficial effects on WM performance and theta and gamma oscillatory power than sham and tDCS in post-stimulation conditions. In contrast to the aforementioned studies, Murphy et al. used the tRNS + DC offset. However, the effectiveness of the tRNS without a DC offset has not been examined, and an online assessment has not been conducted. Moreover, their results conflict with our findings that the tRNS + DC offset had no effect during or after stimulation.

A possible reason that tRNS + DC was not effective in the current study may have been that the complexity of physiological interaction affected the combination of noise intensity and DC offset. Given the results of a previous study [22], a reasonable interpretation may be that this combination lowered the optimal tRNS noise intensity. The possibility of optimal noise intensity has been suggested across various sensory functions, including visual perception, auditory perception, and tactile stimulus detection [69–71], and at least in the present study, the effectiveness of 2 mA tRNS was identified. This indicates that the intensity of tRNS in this study, which targets working memory, is not far off from the optimal noise intensity. Besides, the effectiveness of tRNS 1 mA + DC has been confirmed in a previous study [22]. Thus, our noise intensity in the tRNS + DC offset condition was too high compared with the changed optimal noise intensity, resulting in the degradation of information content from the physiological signal [72]. Another possible reason was the delivery of a polarized current (consistent polarization of the membrane potential), suggesting that the addition of DC offset attenuated the larger effects of tRNS-to-WM-related network functional connectivity, resulting in the failure to improve task performance. However, the neurophysiological mechanisms of tRNS and tRNS + DC offset in the brain network have not yet been clarified, and little is known about the underlying mechanisms of both techniques. The influence of different task-related brain activity patterns, depending on the nature and difficulty of the target task, could be another possible explanation for this inconsistency. Therefore, further research that directly compares the neurophysiological (cortical excitability) and cognitive effects of delivering tRNS with and without a DC offset, including online and post-stimulation assessments, is needed.

Regarding the results showing a significant effect of tRNS during stimulation, but not post-stimulation, the different physiological mechanisms that occurred in the two time periods may have affected the outcomes. Acute online benefits were generated by enhancing the response of neural populations for near-threshold derived from the physiological effects of voltage-gated sodium channel activities [66]; an offline after-effect was involved in a relatively long-lasting GABAergic disinhibition mechanism, working in GABA activity reduction [73] associated with increased activity within facilitatory cortical circuits, which might facilitate neural transmission at the population level [74]. However, both these reports were based on studies targeting the primary motor cortex, not the DLPFC, and should be interpreted with caution. Moreover, strong physiological evidence supporting these differences has not yet been established. Previous reports that examined tRNS effects on WM commonly show decreased benefits after stimulation compared with those during stimulation, and trends using other tES techniques [22, 39, 40].

Regarding the relationship between benefits and basic performance, we found significant differences in the regression line slope between tRNS and sham and between tDCS and sham during stimulation, suggesting that both techniques had performance-dependent effects on WM. In particular, given the significant online effect and greater line slopes generated by the benefits and basic performance in tRNS, tRNS may have had a greater effect on individuals with low online WM performance. A previous study reported that tRNS in healthy adults successfully improved cognitive function and had stronger effects in individuals with lower attention control [30]. These findings provide important insights into understanding the individual variability of tRNS effects on cognitive processes and highlight the potential utility of tRNS as a tool for enhancing cognitive functions, including WM.

This study has some limitations. First, only the short-term effects of tES during one 20 min stimulation session on WM performance were evaluated. Continuous and repeated long-term follow-up assessments are necessary to determine the durability and persistence of any observed improvements. Second, the lack of tRNS standardized protocols, including variations in stimulation parameters such as intensity, duration, and electrode allocation, prevented verification of the optimality of our results. This variability makes it difficult to compare results across studies and determine the optimal parameters for enhancing WM. Third, DBT was used, which is a challenging and complex WM task. Task-related activation patterns vary depending on task content and difficulty in WM. Such variability in WM task types among studies makes it challenging to compare results and draw definitive conclusions regarding tRNS effectiveness. Fourth, precise electrode placement customized for an individual’s brain structure or function was not implemented. The combination of structural magnetic resonance imaging (MRI) and neuronavigation systems significantly improves the precision of electrode placement, allowing for spatially consistent current stimulation. Moreover, functional MRI can precisely measure task-related brain activation, making it an ideal candidate for guiding electrode placement and contributing to a better understanding of tES effectiveness from a neurophysiological perspective. Finally, the generalizability of our findings is limited because they were validated in a healthy population. However, previous neuroimaging studies reported that the DLPFC plays an important role in WM in clinical populations or healthy adults and that the observed effects of tDCS over the left DLPFC were similar in each population; thus, our results may be generalized to include psychiatric and other brain disorders. Further research is required to determine the generalizability of these findings in populations with psychiatric and other brain disorders.

## Conclusions

In conclusion, the results of this study demonstrated significant online effects of tRNS, when administered over the left DLPFC, and greater benefits for healthy individuals with lower WM performance. These findings contribute basic evidence validating tRNS efficacy in clinical populations and suggest that it is a promising tool for improving WM in patients with mild cognitive decline, such as those with brain injury and neurodegenerative and psychiatric disorders.

## Electronic supplementary material

Below is the link to the electronic supplementary material.

Supplementary Material 1

Supplementary Material 2

## Data Availability

The datasets generated and/or analyzed in the current study are available from the corresponding author upon reasonable request.

## Abbreviations

ANOVA: Analysis of variance
CI: Confidence interval
DBT: Dual n-back task
DC: Direct current
DLPFC: Dorsolateral prefrontal cortex
EEG: Electroencephalography
EHI: Edinburgh Handedness Inventory
LMM: Linear mixed models
PASAT: Paced Auditory Serial Addition Task
tDCS: Transcranial direct current stimulation
tES: Transcranial electrical stimulation
tRNS: Transcranial random noise stimulation
WM: Working memory
