# Supplementary material for: Differing effectiveness of transcranial random noise stimulation and transcranial direct current stimulation for enhancing working memory in healthy individuals: a randomized controlled trial
Source: J Neuroeng Rehabil. 2024 Oct 14;21:180. doi: 10.1186/s12984-024-01481-z (PMC11472542; doi:10.1186/s12984-024-01481-z)
Supplement: Supplementary file 1 — Supplementary Material 1 [file 12984_2024_1481_MOESM1_ESM.docx]

**
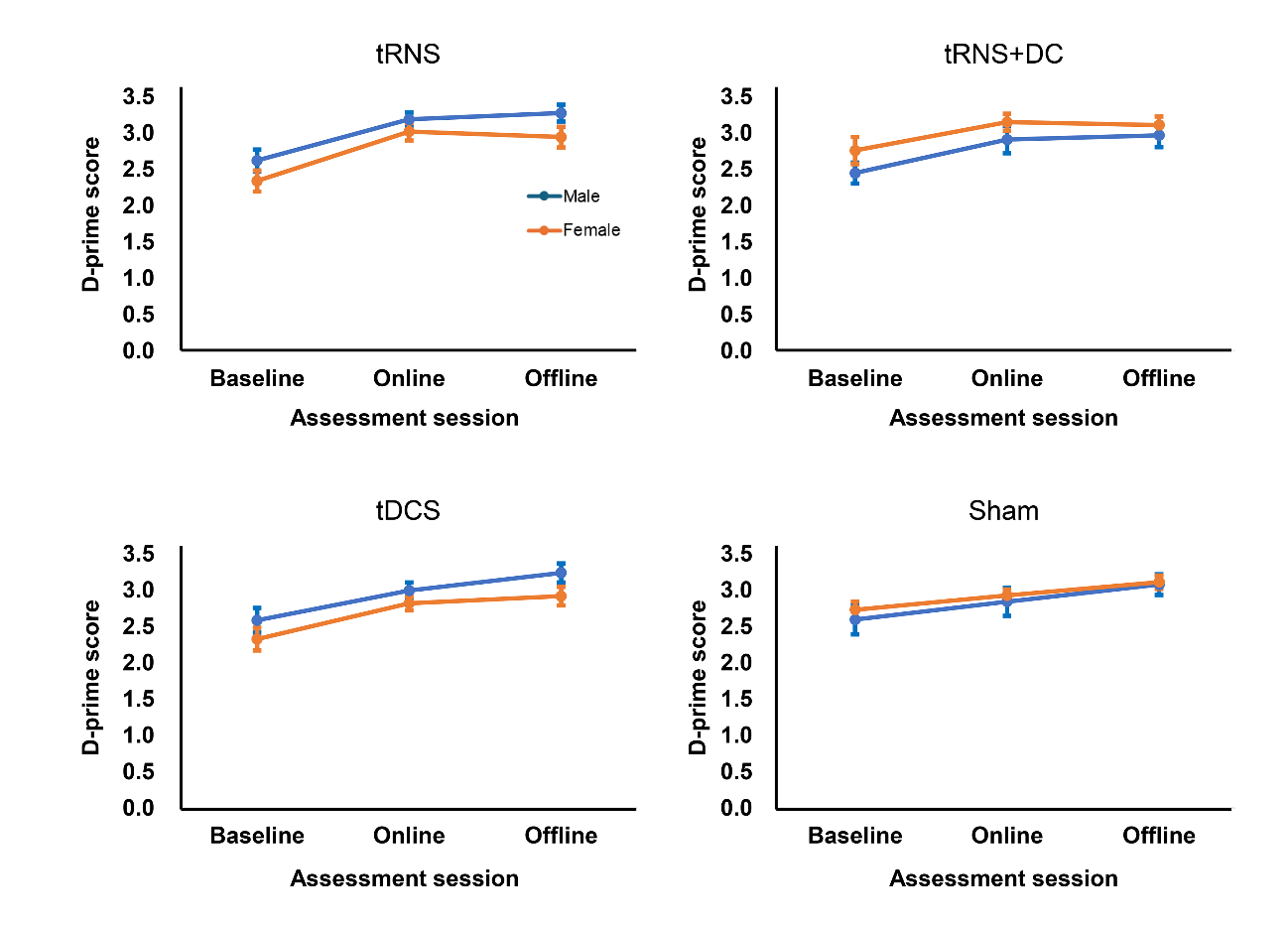
Supplementary Figure 1.** Trends in the d-prime score by sex across groups.

No significant differences were observed in any of the stimulation types.

tRNS: transcranial random noise stimulation, tDCS: transcranial direct current stimulation.


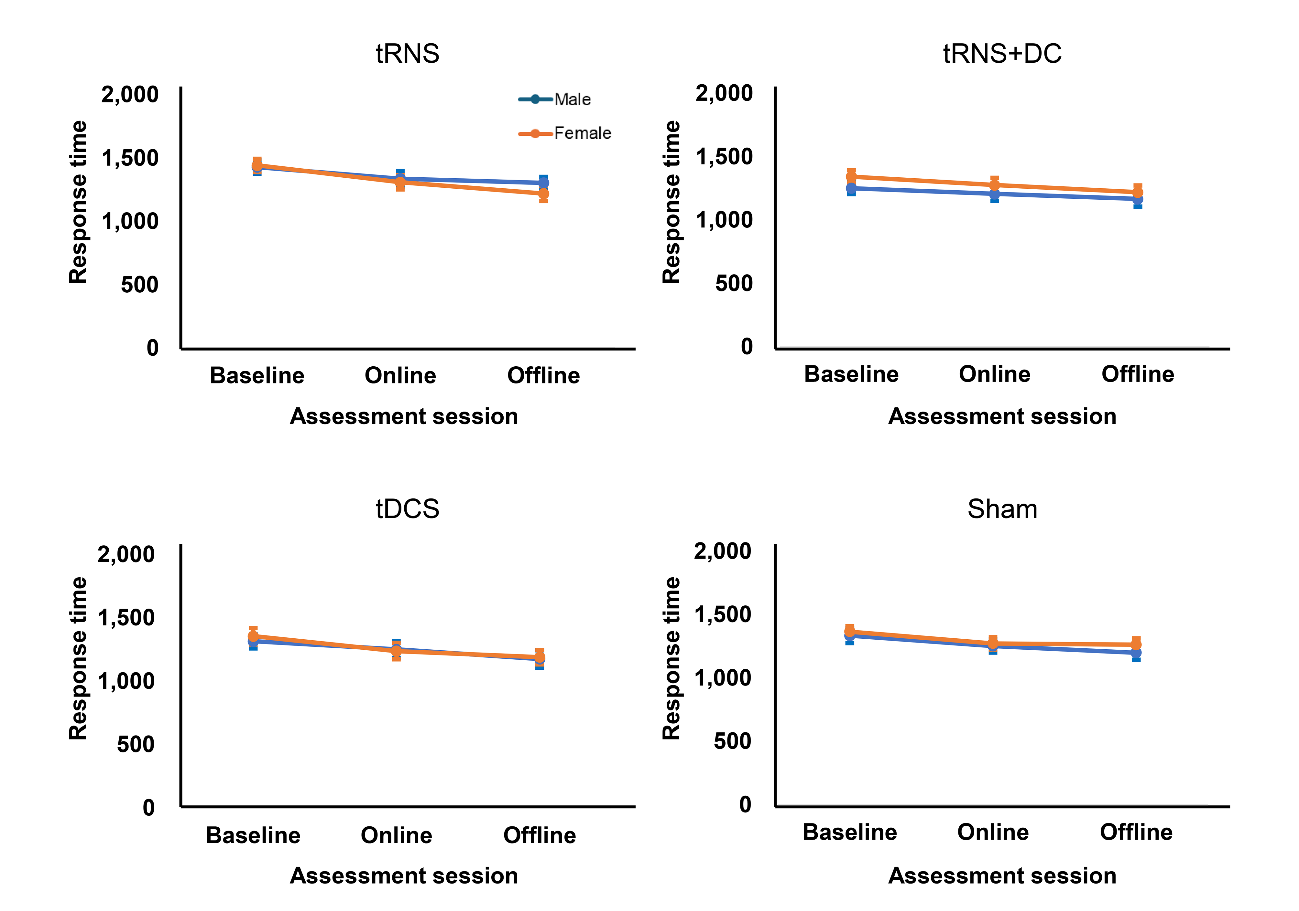


**Supplementary Figure 2.** Trends in response time by sex across groups.

No significant differences were observed in any of the stimulation types.

tRNS: transcranial random noise stimulation, tDCS: transcranial direct current stimulation.
